# SARS-CoV-2 Infection Is Associated with Uncontrolled HIV Viral Load in Non-Hospitalized HIV-Infected Patients from Gugulethu, South Africa

**DOI:** 10.3390/v14061222

**Published:** 2022-06-03

**Authors:** Humaira Lambarey, Melissa J. Blumenthal, Abeen Chetram, Wendy Joyimbana, Lauren Jennings, Marius B. Tincho, Wendy A. Burgers, Catherine Orrell, Georgia Schäfer

**Affiliations:** 1 International Centre for Genetic Engineering and Biotechnology (ICGEB), Cape Town 7925, South Africa; lmbhum001@myuct.ac.za (H.L.); melissa.blumenthal@uct.ac.za (M.J.B.); abeen.chetram@icgeb.org (A.C.); 2 Faculty of Health Sciences, Institute of Infectious Disease and Molecular Medicine (IDM), University of Cape Town, Cape Town 7925, South Africa; marius.tincho@uct.ac.za (M.B.T.); wendy.burgers@uct.ac.za (W.A.B.); catherine.orrell@hiv-research.org.za (C.O.); 3 Department of Integrative Biomedical Sciences, Division of Medical Biochemistry, University of Cape Town, Cape Town 7925, South Africa; 4 Desmond Tutu Health Foundation, Cape Town 7925, South Africa; qaqa.bangani@gmail.com (W.J.); lauren.jennings@hiv-research.org.za (L.J.); 5 Department of Pathology, Division of Medical Virology, University of Cape Town, Cape Town 7925, South Africa; 6 Wellcome Centre for Infectious Diseases Research in Africa, University of Cape Town, Cape Town 7925, South Africa

**Keywords:** HIV, SARS-CoV-2, COVID-19, South Africa, viral load, PLWH, ART

## Abstract

In South Africa, high exposure to SARS-CoV-2 occurs primarily in densely populated, low-income communities, which are additionally burdened by highly prevalent Human Immunodeficiency Virus (HIV). With the aim to assess SARS-CoV-2 seroprevalence and its association with HIV-related clinical parameters in non-hospitalized patients likely to be highly exposed to SARS-CoV-2, this observational cross-sectional study was conducted at the Gugulethu Community Health Centre Antiretroviral clinic between October 2020 and June 2021, after the first COVID-19 wave in South Africa and during the second and beginning of the third wave. A total of 150 adult (median age 39 years [range 20–65 years]) HIV-infected patients (69% female; 31% male) were recruited. 95.3% of the cohort was on antiretroviral therapy (ART), had a median CD4 count of 220 cells/µL (range 17–604 cells/µL) and a median HIV viral load (VL) of 49 copies/mL (range 1–1,050,867 copies/mL). Furthermore, 106 patients (70.7%) were SARS-CoV-2 seropositive, and 0% were vaccinated. When stratified for HIV VL, patients with uncontrolled HIV viremia (HIV VL > 1000 copies/mL) had significantly higher odds of SARS-CoV-2 seropositivity than patients with HIV VL < 1000 copies/mL, after adjusting for age, sex and ART status (*p* = 0.035, adjusted OR 2.961 [95% CI: 1.078–8.133]). Although the cause–effect relationship could not be determined due to the cross-sectional study design, these results point towards a higher risk of SARS-CoV-2 susceptibility among viremic HIV patients, or impaired HIV viral control due to previous co-infection with SARS-CoV-2.

## 1. Introduction

The COVID-19 pandemic in Sub-Saharan Africa (SSA) has unfolded against the backdrop of a substantial and longstanding HIV epidemic. In South Africa, there were an estimated 7.8 million people living with HIV (PLWH) as of 2020, 68% of whom received antiretroviral treatment (ART) [1].

While the morbidity and mortality of the COVID-19 pandemic in SSA has been substantially lower than in the Americas, Europe and Asia [2], South Africa, of all African countries, had the highest COVID-19 related morbidity and mortality [3]. As was the case elsewhere in the world, the strongest risk factor for death from COVID-19 in South Africa was advanced age, which outweighed the risks associated with any other demographic factor or medical condition, such as hypertension, cardiovascular disease, chronic lung disease, obesity, or diabetes [4,5]. However, the high prevalence of HIV in South Africa has been an area of concern from early in the pandemic, but data is conflicting as to whether there was an interplay between the COVID-19 and the HIV/AIDS pandemics. Initial small cohort studies, primarily conducted in North America and Europe, identified no clear evidence for higher SARS-CoV-2 infection rates or adverse disease outcomes for COVID-19 hospitalized PLWH [6,7,8,9,10,11]. Subsequently, larger studies conducted in the UK identified a moderately increased risk of mortality among PLWH after adjusting for age, sex and other comorbidities (aHR 1.50, 95% CI 1.02–2.22) [12], which was confirmed by studies from the Western Cape province of South Africa (aOR 2.14, 95% CI 1.70–2.70) [13] and from all provinces of South Africa (aOR 1.34, 95% CI 1.27–1.43) [5]. Here, patients with low CD4 count (<200 cells/µL) and uncontrolled HIV infection (VL > 1000 copies/mL) had a more severe clinical course than HIV negative patients. Moreover, the WHO Global COVID-19 Clinical Data Platform identified HIV as an independent risk factor for severe critical illness at hospital admission (aOR 1.13, 95% CI 1.09–1.17) and in-hospital mortality (aOR 1.30, 95% CI 1.24–1.36) [14]. These studies also showed that despite effective ART, HIV infection increased the risk of COVID-19 related mortality [5,12,13,14], with patients not on ART being even more likely to die in hospital than those on ART [5]. While ART seems to confer some protection against severe COVID-19, the potential relationship of ART with COVID-19 outcomes remains controversial. Some studies suggest protective effects of tenofovir for SARS-CoV-2 infection and reduced hospitalization and mortality compared to other therapies [13,15,16,17] but there might be selection bias as most patients with co-morbidities, such as renal failure, do not take tenofovir. 

The first wave of the COVID-19 pandemic peaked in South Africa in July 2020, followed by a second wave, which peaked in January 2021, a third wave peaking in July 2021 and a fourth wave, which peaked in January 2022. It was suspected that undetected widespread transmission of SARS-CoV-2 occurred early in the pandemic, particularly in communities with limited social distancing. Indeed, low-income communities were at a higher risk of SARS-CoV-2 infection and of COVID-19 related mortality, and tended to be worse affected in the first wave with relative protection in the second wave [18]. Several seroprevalence studies from South Africa demonstrated that high SARS-CoV-2 seroprevalence was associated with informal housing, living in a low-income household and having a low-earning occupation [19]. While poverty and HIV infection seem to negatively impact COVID-19 severity and mortality, studies found that HIV infection was not associated with increased risk of SARS-CoV-2 susceptibility after adjusting for confounding factors such as increased exposure to both infections due to socioeconomic vulnerability and lack of social distancing [20].

Most studies published to date have focused on the impact of HIV infection on COVID-19 severity among hospitalized patients, with death as the outcome parameter. We therefore asked whether there were any associations between previous SARS-CoV-2 infection (as retrospectively assessed by seroconversion) and HIV-related and clinical parameters in non-hospitalized PLWH. HIV-infected patients on ART with a moderately low recently recorded CD4 count (<350 cells/µL) attending an HIV clinic in an informal setting outside Cape Town, South Africa, were recruited to this study after the first COVID-19 wave and during the second and beginning of the third wave. SARS-CoV-2 seroprevalence was >70%, confirming previously reported high viral transmission typical for informal settings [19]. We identified a significant association between uncontrolled HIV viremia (VL > 1000 copies/mL) and SARS-CoV-2 seropositivity. While this correlation does not prove causation, our results point towards a higher risk of SARS-CoV-2 susceptibility among PLWH. Alternatively, HIV viral control could have been impaired by previous co-infection with SARS-CoV-2.

## 2. Materials and Methods

### 2.1. Study Cohort

A cohort of 150 non-hospitalized adult HIV-infected patients presenting for routine HIV treatment at the Gugulethu Community Health Centre Antiretroviral clinic (Desmond Tutu HIV Centre, UCT), South Africa, a public-sector antiretroviral delivery site, were enrolled in this study between October 2020 and June 2021. The start of patient recruitment coincided with the decline of SARS-CoV-2 infections from the first COVID-19 wave, spanning the second and beginning of the third wave.

Patients were selected if their latest CD4 count was <350 cells/µL, according to their clinical files. The demographic and clinical characteristics of all patients included in this study are presented in Table 1. The study was conducted according to the declaration of Helsinki, conformed to South African Good Clinical Practice guidelines, and was approved by the University of Cape Town’s Health Sciences Research Ethical Committee (HREC 134/2020). All participants provided written informed consent.

### 2.2. Clinical Data

Clinical and demographic details were collected at enrolment, including any self-reported symptoms at presentation. Peripheral blood was analyzed by the National Health Laboratory Services (NHLS) on the day of enrolment for absolute CD4 count using the Aquios PLG panel (CD45-FITC/CD4 PE monoclonal antibodies) together with an Aquios CL Flow cytometer (Beckman Coulter), as well as for HIV VL using the ALINITY mHIV-1 ASSAY (Abbott Molecular Inc., Des Plaines, IL, USA), following standard operating procedures. Additional tests to determine sodium, creatinine, albumin, alanine transaminase (ALT), C-reactive protein (CRP), hemoglobin, and full blood count and differential cell count were also performed by the NHLS. All data are herein reported using NHLS defined thresholds and ranges as previously determined to be applicable for the general population of South Africa. Information on ART was obtained from pharmacy records. SARS-CoV-2 seroprevalence was determined by in-house ELISA (see “SARS-CoV-2 serology”). All clinical, demographic and experimental data were recorded and stored on an electronic REDCap database [21], hosted by the University of Cape Town.

### 2.3. SARS-CoV-2 Serology

The ELISA protocol used to determine SARS-CoV-2 serology was adapted from Makatsa et al., 2021 [22]. Briefly, 96-well plates (Nunc, Thermo Fisher) were coated with 50 μL of purified RBD and S1 proteins (Cape Bio Pharms, Cape Town, South Africa) at a concentration of 2 μg/mL at 4 °C overnight. Plates were washed five times using PBS with 0.1% Tween20 (PBS-T) and then incubated in blocking buffer (1% casein in 1% PBS-T) at room temperature for 1 h. The blocking buffer was discarded and 100 μL of plasma (1:50 dilution in 0.5% casein in 1% PBS-T) was added to the plate at room temperature for 2 h. Thereafter, the plates were washed five times (as before) and incubated at room temperature for 1 h with either goat anti-human IgG (Fc-specific) peroxidase conjugate (1:5000; IgG-HRP, Sigma), goat anti-human IgM peroxidase conjugate (1:2000; IgM-HRP, Sigma) or goat anti-human IgA (α-chain specific), F(ab’)2 fragment peroxidase conjugate (1:5000; IgA-HRP, Sigma). To develop the plates, 100 μL O-phenylenediamine dihydrochloride (OPD, Sigma) was added to the plates at room temperature for 12 min and the reaction stopped with 50 μL 3 M hydrochloric acid (HCl, Sigma). Plates were read immediately at 490 nm using a Glomax plate reader (Promega). A cut-off for positivity for these patient samples was set at 2SD above the mean optical density (OD) of 30 pre-pandemic samples [22] for each plate. Adjusted OD values were then normalized to cut-off which was set as one.

### 2.4. Statistical Analysis

Statistical tests were performed using SPSS version 25 (IBM Corp, New York, NY, USA). To assess the independent associations of previous SARS-CoV-2 infection (as defined by positive IgG serology to either RBD or S1) in HIV positive patients, binomial logistic regression was performed, controlling for the relevant demographic and clinical parameters as indicated. Continuous variables were transformed, where appropriate, to approximate normal distributions. Non-parametric Spearman Rank tests were used for all correlations. HIV VL was treated both as a categorical variable (<1000 copies/mL vs. >1000 copies/mL) and a continuous variable and assessed for association with positive SARS-CoV-2 serology using the χ^2^, Fisher’s exact, or Mann–Whitney test, as appropriate. *p*-values are two-tailed and were considered significant if <0.05.

## 3. Results

With the intention to characterize a non-hospitalized non-vaccinated patient cohort with regards to HIV-related clinical parameters after high exposure to SARS-CoV-2, adult HIV-infected patients with low CD4 count were enrolled consecutively in this study after the first wave of SARS-CoV-2 infections, continuing during the second and beginning of the third wave. No patients had received COVID-19 vaccinations yet. 

### 3.1. Clinical Characteristics of the Study Participants

Eligible patients were defined as those with confirmed HIV infection with the last recorded CD4 count of <350 cells/µL. This threshold ensured the inclusion of immunosuppressed patients without restricting the study entirely to advanced HIV disease (as defined by CD4 count < 200 cells/µL [23]). All baseline demographic information of the patient cohort (*n* = 150) together with clinical characteristics relevant to this study are listed in Table 1.

**Figure 1:**
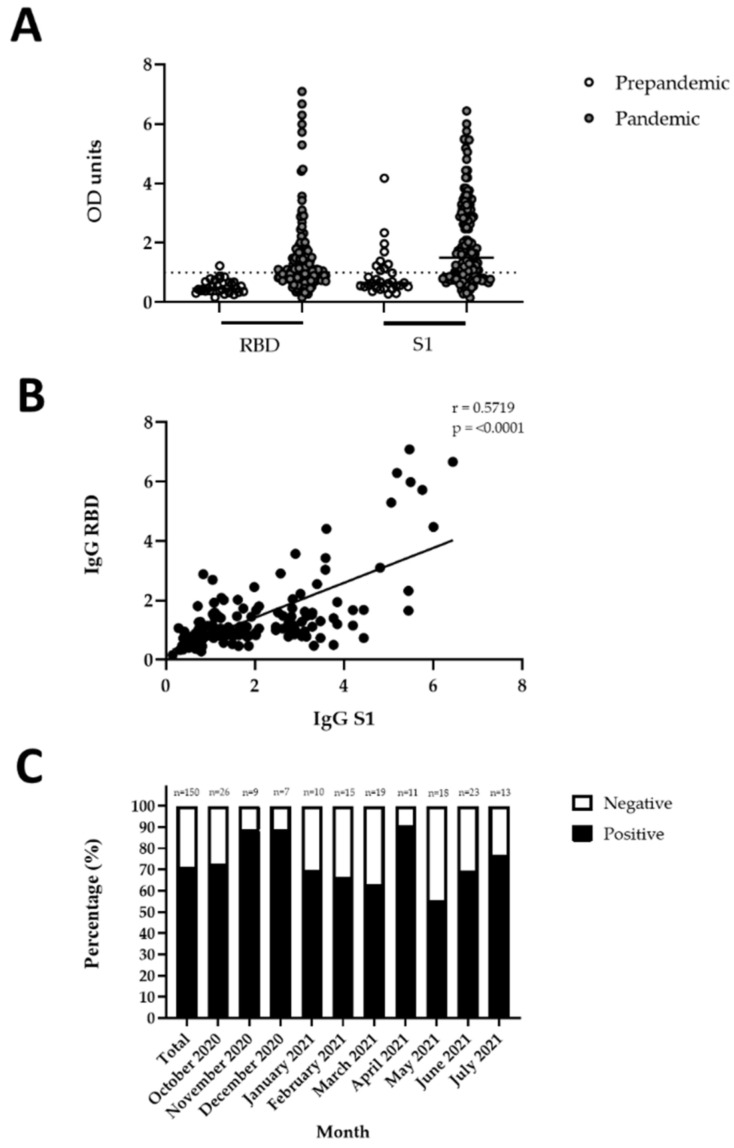
Seroprevalence of SARS-CoV-2 in patient cohort (*n* = 150): (**A**) Detection of SARS-CoV-2 RBD- and S1-specific IgG antibodies in the study participants’ plasma. A total of thirty pre-pandemic patient samples [22] served as control. Results are represented by the OD units of each isotype, adjusted to the cut-off value of each individual plate and then normalized to the cut-off, which was set as one (indicated by the dotted line). The cut-off was determined by the mean OD + 2SD of the pre-pandemic samples; (**B**) The correlation between IgG responses to SARS-CoV-2 RBD and S1 antigens. Statistical analyses were performed using a non-parametric Spearman Rank correlation; (**C**) Timeline of SARS-CoV-2 IgG antibody detection per month over the course of the recruitment period from October 2020 to June 2021. Data is represented as a percentage (positive or negative) for the patient cohort. The total number of patients is indicated above the bars. A patient was considered to be positive for SARS-CoV-2 infection if either RBD or S1 responses were above our calculated cut-off values for the IgG antibody.

Briefly, 30.9% of the patients were men and 69.1% women with a median age of 39 years (range 20–65). All patients were HIV infected with a median HIV VL at the time of recruitment of 49 copies/mL (range 1–1050867), of whom 73.4% had an HIV VL < 1000 copies/mL. The actual CD4 counts at the time of recruitment were re-determined and slightly differed from the last clinical records with an average of 220 cells/µL (range 17–604), representing 80% of the cohort below normal range (CD4 count < 332 cells/µL as per NHLS definition). Patient medical records indicated that the average time since HIV diagnosis was 1305 days (range 0–10303) and the average time since start of ART was 527 days (range 0–5238). Almost all patients (95.3%) received ART at the time of recruitment, with the majority on first-line regimens consisting of tenofovir/emtricitabine/efavirenz (TEE) (44.8%) and tenofovir/lamivudine/dolutegravir (TLD) (33.6%), respectively. The majority of the patient cohort were assigned a WHO clinical stage of HIV disease of one (57.7%), followed by stage two (24.1%), stage three (16.8%) and stage four (1.5%). More than two thirds of the patients (70.7%) showed a positive SARS-CoV-2 antibody response as assessed by an in-house ELISA (Figure 1A), indicating a previous infection as none of the patients were vaccinated against COVID-19 at the time of recruitment. However, we had neither information on the actual date of acute SARS-CoV-2 infection, nor on the date of symptom onset (if any) or COVID-19 disease severity, limiting the analysis to seropositivity only, assuming that the infection occurred during the first or second wave of COVID-19. Interestingly, there was a very high percentage of SARS-CoV-2 seropositive patients early on during the recruitment process, reflecting infection rates of the first COVID-19 wave, which did not significantly change over the course of patient enrolment (Figure 1C).

Although we have tested the patient sera for IgG, IgA and IgM antibodies following a robust in-house protocol [22], only IgG positive results (RBD or S1) were included in the final analysis (Figure 1A). Both RBD and S1 are highly immunogenic protein fragments and have been used in several applications for the serological testing of SARS-CoV-2 seropositivity in patient plasma samples. As expected, the responses to RBD were more stringent and produced less cross-reactivity in the pre-pandemic cohort than S1 responses, which showed some cross-reactive samples above threshold. However, since responses to RBD and S1 showed good correlation in a Spearman Rank test (Figure 1B, r = 0.5719, *p* < 0.0001), we considered a patient result positive if an OD value above cut-off to either IgG RBD or S1 was detected. Although IgM and IgA immune responses to RBD and S1 were specific to SARS-CoV-2 when compared to a pre-pandemic cohort (Figure S1A), IgG responses are known to persist longer and stay at relatively high levels post-illness onset, compared to IgA and IgM responses, which wane soon after they peak [24,25], making IgG the most suitable immunoglobulin for determining seroprevalence in a cohort of unknown date of acute infection (Table S1). This was also confirmed by correlation analyses, which showed moderate, albeit significant, Spearman Rank correlation between IgG and IgA responses to RBD (r = 0.3613, *p* = 0.0001), and IgG and IgM responses to RBD (r = 0.2812, *p* = 0.0005), as shown in Figure S1B. There was less to no correlation between IgG and IgA responses to S1 (r = 0.2209, *p* = 0.0066) and IgG and IgM responses to S1 (r = 0.9433, *p* = 0.2509), as shown in Figure S1C. To avoid false-negative results due to the waning of IgM or IgA below threshold levels, we included only IgG results and excluded IgA and IgM serologies in all downstream analyses. 

### 3.2. Association of Clinical Parameters with SARS-CoV-2 Seroconversion

Upon recruitment, blood samples of all study participants were extensively characterized with regards to HIV-related and clinical parameters (Table 2). Patients also self-reported on selected symptoms at presentation (Table S2).

**Figure 2:**
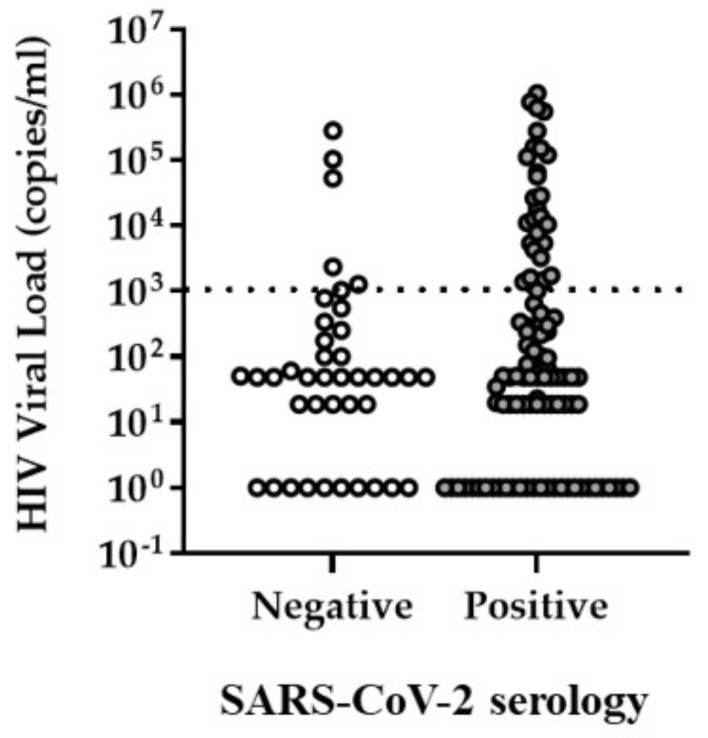
Distribution of HIV VL in SARS-CoV2 seropositive and negative patients. The dotted line indicates the threshold (HIV VL = 1000 copies/mL) used for determining uncontrolled HIV viremia.

By univariate analysis, we detected a significant association between SARS-CoV-2 serology status and elevated (>1000 copies/mL) HIV VL (31.4% versus 14.6% uncontrolled HIV VL in SARS-CoV-2 seropositive versus seronegative patients, *p* = 0.04, Figure 2), sodium concentration (45.6% below normal range in SARS-CoV-2 seropositive (range 128–144 mmol/L) versus 27.9% in seronegative patients (range 134–143 mmol/L), *p* = 0.047), mean corpuscular hemoglobin as a measure for oxygen (8.7% versus 0% below normal range in SARS-CoV-2 seropositive versus seronegative patients, *p* = 0.048), monocyte count (0% versus 6.8% above normal range in SARS-CoV-2 seropositive versus seronegative patients, *p* = 0.029), CD45+ white cell count (0% versus 4.8% above normal range in SARS-CoV-2 seropositive versus seronegative patients, *p* = 0.041) and CD4 percentage of lymphocytes (96.1% versus 83.3% below normal range in SARS-CoV-2 seropositive versus seronegative patients, *p* = 0.014). Interestingly, in addition to uncontrolled HIV VL, hyponatremia and anemia are indicative of HIV disease severity [26,27], as well as of adverse COVID-19 disease outcome [5,13,28,29]. 

No other significant associations were identified, although there was a trend of longer time since HIV diagnosis (1610 days versus 709 days, *p* = 0.082) in SARS-CoV-2 seropositive patients. Furthermore, there were slightly more SARS-CoV-2 seroconverted patients with red blood cell count below normal range (40.8% versus 22.7%, *p* = 0.081). 

We did not identify a significant impact of the individual ART regimen on SARS-CoV-2 infection, although there were slightly more SARS-CoV-2 seronegative patients receiving TDF, FTC, EFV (TEE), one of the most commonly prescribed first-line ART, than SARS-CoV-2 seropositive patients (54.8% versus 40.6%). Interestingly, more patients on the less commonly prescribed ART (indicated by numbers 3–12 in Table 2) were SARS-CoV-2 seropositive than seronegative. However, when all patients on these less common treatments (*n* = 5 (11.9%) SARS-CoV-2 seronegative and *n* = 26 (25.7%) SARS-CoV-2 seropositive) were compared to all patients on TEE and TLD, no statistical significance was detected (*p* = 0.1386), data not shown.

Further assessment of relevant parameters that differed between SARS-CoV-2 seropositive versus seronegative patients by multivariate analysis revealed that SARS-CoV-2 antibody response was associated with HIV VL control after adjusting for age, sex, ART status, CD45+ white cell count, mean corpuscular hemoglobin and sodium (Table 3, Model A, *p* = 0.043, adjusted OR 2.915 [95% CI: 1.035–8.210]). To avoid overfitting the model due to a limited sample size, variables that were not significant in model A were removed and a stripped-down logistic regression is presented, confirming that uncontrolled HIV VL was associated with SARS-CoV-2 seropositivity after adjusting for age, sex, and ART status (Table 3, Model B, *p* = 0.035, adjusted OR 2.961 [95% CI: 1.078–8.133]).

## 4. Discussion

Although it is debated whether HIV infection influences SARS-CoV-2 susceptibility, co-infection with SARS-CoV-2 may have currently unknown short- or longer-term impacts on HIV and/or COVID-19 following acute infection in PLWH on effective ART. Our study revealed significant correlation between SARS-CoV-2 seropositivity and uncontrolled HIV VL (>1000 copies/mL) in an HIV-infected non-hospitalized patient cohort, the majority of whom were on ART (95.3%, average time 527 days since start of ART) and self-reportedly asymptomatic (80%) at the time of recruitment. Since this association study cannot prove causation due to the cross-sectional study design, our results suggest either higher SARS-CoV-2 infection susceptibility in HIV-infected viremic patients, or long-term effects of previous SARS-CoV-2 infection on HIV VL. Indeed, a recent study reported a higher percentage of PLWH on ART with low-level plasma HIV RNA following COVID-19 disease compared to pre-pandemic patients, suggesting that COVID-19 may lead to lasting perturbations of immune functions affecting the natural course of HIV infection [30]. However, it is unknown whether these low-level viremic episodes may have long-term impacts on HIV dynamics and viral immune responses.

For this study, we recruited patients with last recorded CD4 counts of <350 cells/µL, which we considered to be a threshold indicative of immunosuppression. Studies from South Africa have indicated that hospitalized patients with low CD4 count (<200 cells/µL) and uncontrolled HIV infection had more severe COVID-19 disease than HIV negative patients [5]. However, we did not see a correlation between low CD4 count and SARS-CoV-2 seropositivity when categorized into very low (<200 cells/µL) and low (200–332 cells/µL), suggesting that susceptibility to SARS-CoV-2 infection is not correlated with immunosuppression. 

Although we cannot exclude the possibility that HIV viremia and low CD4 count affect seropositivity for SARS-CoV-2, it has been reported that HIV-associated parameters (HIV plasma viral load, CD4 and CD8 cell counts, and CD4:CD8 ratio) were not significantly associated with SARS-CoV-2 antibody losses in PLWH on stable ART from Durban, KwaZulu-Natal, South Africa, recruited after the first COVID-19 wave, between June 2020 and November 2020 [31].

Despite some reports in the literature, our study did not identify any ART regime (even containing tenofovir [13,15,16,17]) to be protective against SARS-CoV-2 infection. Most patients in this study had been on ART long before the SARS-CoV-2 outbreak (average 527 days at time of recruitment), and therefore should have been protected against infection if any of the ART drugs had been effective. Our study did not support this hypothesis, given the >70% SARS-CoV-2 seroprevalence after the first COVID-19 wave when this study commenced. Moreover, studies on hospitalized COVID-19 patients revealed that despite effective ART, HIV infection even slightly increased the risk of COVID-19 related mortality, i.e., HIV suppression on ART did not decrease the mortality risk [5,12,13,14].

This study was limited to the assessment of SARS-CoV-2 seropositivity measuring IgG responses to either S1 or RBD. Although we assumed that IgG responses persist at relatively high levels over long periods of time after acute disease onset (unlike IgA and IgM responses) [24,25], we cannot exclude that some patients previously infected with SARS-CoV-2 had antibody levels below the detection threshold. Nevertheless, the percentage of SARS-CoV-2 seropositive patients at the beginning of the recruitment process in October 2020 indicates very high community exposure and a severe first COVID-19 wave. This high level of SARS-CoV-2 seropositivity did not significantly change over the course of this study, confirming continuously high exposure and little waning of the IgG response to SARS-CoV-2. The high exposure to SARS-CoV-2 early on in the pandemic, even with very strict lockdown measures in place [32], supports observations that low-income communities were particularly vulnerable to SARS-CoV-2 infections as they tend to be overcrowded and denser, rely more on public transport, are less able to implement and maintain social distancing and non-pharmaceutical interventions [18]. Furthermore, the disruption of HIV prevention and treatment services in connection with the COVID-19 related national lockdown [33], could have affected onset of HIV viremia, although this would have affected all study participants in the same way, regardless of SARS-CoV-2 exposure. 

While there was neither information on the date of acute SARS-CoV-2 infection nor on severity of the infection (if symptomatic), this study nevertheless suggests that patients with increased HIV VL should be more closely screened for comorbidities that are known to increase the risk of long-term COVID-19-related morbidity. Indeed, there is evidence from several independent South African datasets that HIV viremia and/or low CD4 count in PLWH may be associated with longer SARS-CoV-2 shedding [34,35,36]. However, these studies are limited by relatively small numbers of participants with advanced disease; therefore, a precise definition of the population at risk for persistent SARS-CoV-2 infection is not yet possible.

Our study focused on non-hospitalized unvaccinated HIV-infected patients with potentially high exposure to SARS-CoV-2. Although our data are derived from a single-center study and cannot be generalized to the entire HIV-infected population in the country, we believe that our results reflect an approximate representation of SARS-CoV-2 distribution in HIV-infected non-hospitalized individuals attending an ART center in a low-income setting in South Africa. HIV treatment programs in the public sector have been substantially scaled up in the last decade, which resulted in significantly improved rates of viral suppression in HIV-infected individuals. In the Western Cape province, an estimated 90% of PLWH enrolled in public sector services were virally suppressed [37], while a study conducted in KwaZulu-Natal reported 94.5% of those on ART being virally suppressed [38]. Inadequate adherence to ART has been identified as the most common barrier to sustained viral suppression, as well as acquired and transmitted drug resistance and concurrent use of alternative treatments [39]. The interruptions of HIV care programs due to the COVID-19 pandemic [33] could also have led to increased rates of patients displaying an HIV VL > 1000 copies/mL. Our study therefore supports earlier reports in the field with regards to recommended implications for public health responses following the COVID-19 pandemic, such as enhanced monitoring/surveillance for chronic infections in viremic PLWH, optimization of virological suppression of HIV to avoid chronic SARS-CoV-2 infection, and integration of COVID-19 vaccination into HIV services to ensure full vaccination of priority groups [40].

Despite effective ART, a subset of PLWH may continue to experience persistent immune dysfunction and inflammation [41], which may modulate the risk of long-term COVID-19 related morbidity including uncontrolled HIV viremia and SARS-CoV-2 persistence [34,35,36]. These inadequate immune responses could in turn drive viral evolution and the emergence of variants [42]. Current HIV care cascades should therefore be strengthened and ART regimen individually reassessed to address uncontrolled HIV VL even in the absence of immunosuppression in the current era of global SARS-CoV-2 exposure.

## Figures and Tables

**Table 1:** Selected baseline demographic and clinical characteristics of the patient cohort (*n* = 150). All patients were HIV positive adults and were recruited with a last known CD4 count < 350 cells/µL. Data are presented as number and percentage of total or median and range as appropriate. Participants with missing data are excluded per characteristic.

Characteristic		*n* (%) or Median (Range)
Sex	Female	104 (69.3)
	Male	46 (30.7)
Age, years		39 (20–65)
Weight, kgs		69 (34–150)
Time since HIV diagnosis, days		1305 (0–10303)
HIV VL, copies/mL		49 (1–1050867)
HIV VL	<1000 copies/ml	105 (73.4)
	>1000 copies/ml	38 (26.6)
Last known CD4 at time of recruitment ^1^, cells/µL		154 (3–347)
Absolute CD4, cells/µl		220 (17–604)
Absolute CD4	Within normal range ^2^ (332–1642 cells/µL)	29 (20.0)
	Below normal range ^2^ (< 332 cells/µL)	116 (80.0)
WHO clinical stage of HIV disease	1	79 (57.7)
	2	33 (24.1)
	3	23 (16.8)
	4	2 (1.5)
Receiving ART ^3^	Yes	143 (95.3)
	Defaulted	7 (4.7)
Time since ART start ^3^, days		527 (0–5238)
ART regimen ^3^	TDF, FTC, EFV (TEE)	64 (44.8)
	TDF, 3TC, DTG (TLD)	48 (33.6)
	ABC, 3TC, EFV	2 (1.4)
	ABC, 3TC, NVP	1 (0.7)
	AZT, 3TC, LPV/r	9 (6.3)
	TDF, FTC, LPV/r	6 (4.2)
	ABC, 3TC, LPV/r	1 (0.7)
	TDF, FTC, ATV/r	2 (1.4)
	ABC, 3TC, DTG	3 (2.1)
	AZT, 3TC, DTG	2 (1.4)
	ABC, 3TC, ATV/r	4 (2.8)
	TDF, FTC, EFV, LPV/r	1 (0.7)
SARS-CoV-2 antibody ^4^	Negative	44 (29.3)
	Positive	106 (70.7)
SARS-CoV-2 vaccinated		0 (0%)

^1^ Data retrieved from patient medical records reflecting last known result at time of recruitment. ^2^ Range as per NHLS definition. ^3^ Data collated from pharmacy records. ^4^ SARS-CoV-2 antibody positivity detected by ELISA to IgG RBD and S1 (“Negative” indicates that patient sample has an OD value below the assay cut-off for both IgG RBD and S1 ELISAs). Abbreviations: ART, antiretroviral therapy; HIV, human immunodeficiency virus; VL, viral load; FTC, Emtricitabine; EFV, Efavirenz; TDF, Tenofovir; 3TC, Lamivudine; DTG, Dolutegravir, AZT, Zidovudine; LPV/r, Lopinavir/Ritonavir; ABC, Abacavir; ATV/r, Atazanavir/Ritonavir; NVP, Nevirapine; TEE, TDF/FTC/EFV; TLD, TDF, 3TC, DTG.

**Table 2:** Univariate analysis comparing clinical and virological parameters between patients who were negative and positive for SARS-CoV-2 antibodies. All patients (*n* = 150) were HIV positive adults and were recruited with a last known CD4 count < 350 cells/µL. SARS-CoV-2 antibody positivity was detected by ELISA to IgG RBD and S1 (“Negative” indicates that a patient sample had an OD value below the assay cut-off for both IgG RBD and S1 ELISAs). Data are presented as number and percentage of total or median and range as appropriate. Participants with missing data are excluded per characteristic. *p*-values are by Mann–Whitney U test for continuous variables and Chi-square test or Fisher’s Exact test for categorical variables as appropriate. *p* < 0.05 is considered significant and indicated with *.

Parameter			SARS-CoV-2 Antibody Negative (*n* = 44) N (%) or Median (Range)	SARS-CoV-2 Antibody Positive (*n* = 106) N (%) or Median (Range)	*p*-Value
**Demographic information**					
Age, years			39 (27–58)	38 (20–65)	0.898
Sex	Female		29 (65.9%)	74 (70.5%)	0.582
	Male		15 (34.1%)	31 (29.5%)	
Weight, kgs			69.5 (34.0–102.0)	69.0 (41.2–150.0)	0.773
**HIV-related parameters**					
Time since HIV diagnosis, days			709 (0–9993)	1610 (0–10303)	0.082
HIV VL, copies/mL			49 (1–283875)	50 (1–1050867)	0.411
HIV VL	< 1000 copies/mL		35 (85.4%)	70 (68.6%)	0.04 *
	> 1000 copies/mL		6 (14.6%)	32 (31.4%)	
Last known CD4 at time of recruitment ^1^, cells/µL			171 (3–336)	147 (4–347)	0.607
Absolute CD4, cells/µL			222 (32–589)	209 (17–604)	0.925
Absolute CD4	Low (200–332 cells/µL)		14 (33.3%)	39 (37.9%)	0.740
	Very low (< 200 cells/µL)		18 (42.9%)	45 (43.75)	
WHO clinical stage of HIV disease	1		28 (68.3%)	51 (53.1%)	0.174
	2		5 (12.2%)	28 (29.2%)	
	3		7 (17.1%)	16 (16.7%)	
	4		1 (2.4%)	1 (1.0%)	
Receiving ART ^3^	Yes		42 (95.5%)	101 (95.3%)	1.00
	Defaulted		2 (4.5%)	5 (4.7%)	
Time since ART start ^3^, days			527 (0–5238)	526 (0–5064)	0.597
ART regimen ^3^	1	TDF, FTC, EFV (TEE)	23 (54.8%)	41 (40.6%)	0.098
	2	TDF, 3TC, DTG (TLD)	14 (33.3%)	34 (33.7%)	
	3	ABC, 3TC, EFV	0 (0.0%)	2 (2.0%)	
	4	ABC, 3TC, NVP	1 (2.4%)	0 (0.0%)	
	5	AZT, 3TC, LPV/r	0 (0.0%)	9 (8.9%)	
	6	TDF, FTC, LPV/r	0 (0.0%)	6 (5.9%)	
	7	ABC, 3TC, LPV/r	0 (0.0%)	1 (1.0%)	
	8	TDF, FTC, ATV/r	0 (0.0%)	2 (2.0%)	
	9	ABC, 3TC, DTG	2 (4.8%)	1 (1.0%)	
	10	AZT, 3TC, DTG	0 (0.0%)	2 (2.0%)	
	11	ABC, 3TC, ATV/r	1 (2.4%)	3 (3.0%)	
	12	TDF, FTC, EFV, LPV/r	1 (2.4%)	0 (0.0%)	
**Laboratory blood analysis**					
Sodium, mmol/L			137.00 (134.00–143.00)	136.00 (128.00–144.00)	0.026 *
Sodium	Within normal range (136–145 mmol/L) ^2^		31 (72.1%)	56 (54.4%)	0.047 *
	Below normal range (<136 mmol/L) ^2^		12 (27.9%)	47 (45.6%)	
Creatinine, µmol/L			68.00 (47.00–124.00)	67.50 (34.00–109.00)	0.562
Creatinine	Within normal range (F:49–90 µmol/L, M: 64–104 µmol/L) ^2^		37 (86.0%)	86 (82.7%)	0.771
	Below normal range (F: <49 µmol/L, M: <64 µmol/L) ^2^		4 (9.3%)	14 (13.5%)	
	Above normal range (F: >90 µmol/L, M: >104 µmol/L) ^2^		2 (4.7%)	4 (3.8%)	
Albumin, g/L			43.00 (32.00–51.00)	42.00 (28.00–55.00)	0.214
Albumin	Within normal range (35–52 g/L) ^2^		42 (97.7%)	91 (88.3%)	0.186
	Below normal range (<35 g/L) ^2^		1 (2.3%)	9 (8.7%)	
	Above normal range (>52 g/L) ^2^		0 (0.0%)	3 (2.9%)	
Alanine transaminase, (IU/L)			23.00 (8.00–193.00)	20.50 (5.00–67.00)	0.194
Alanine transaminase	Within normal range (F: 7–35 IU/L, M: 10–40 IU/L) ^2^		38 (88.4%)	88 (86.3%)	0.651
	Below normal range (F: <7 IU/L, M: <10 IU/L) ^2^		0 (0.0%)	2 (2.0%)	
	Above normal range (F: >35 IU/L, M: >40 IU/L) ^2^		5 (11.6%)	12 (11.8%)	
C-reactive protein, mg/L			4.00 (1.00–347.00)	4.00 (1.00–82.00)	0.805
C-reactive protein	Within normal range (<10 mg/L) ^2^		34 (79.1%)	74 (71.2%)	0.323
	Elevated(>10 mg/L) ^2^		9 (20.9%)	30 (28.8%)	
White cell count, ×10^9^/L			5.06 (2.53–11.61)	4.91 (2.73–8.77)	0.70
White cell count	Within normal range (F: 3.9–12.6 × 10^9^/L, M: 3.92–10.4 × 10^9^/L) ^2^		33 (75.0%)	81 (78.6%)	0.628
	Below normal range (F: <3.9 × 10^9^/L, M: <3.92 × 10^9^/L) ^2^		11 (25.0%)	22 (21.4%)	
Red cell count, ×10^12^/L			4.14 (3.18–5.55)	4.14 (3.08–5.51)	0.460
Red cell count	Within normal range (F: 3.8–4.8 × 10^12^/L, M: 4.5–5.5 × 10^12^/L) ^2^		33 (75.0%)	57 (55.3%)	0.081
	Below normal range (F: <3.8 × 10^12^/L, M: <4.5 × 10^12^/L) ^2^		10 (22.7%)	42 (40.8%)	
	Above normal range (F: >4.8 × 10^12^/L, M: >5.5 × 10^12^/L) ^2^		1 (2.3%)	4 (3.9%)	
Haemoglobin, g/dL			12.70 (8.70–16.60)	12.50 (6.00–17.00)	0.269
Haemoglobin	Within normal range (F: 12–15 g/dL, M: 13–17 g/dL) ^2^		26 (59.1%)	64 (62.1%)	0.729
	Below normal range (F: <12 g/dL, M: <13 g/dL) ^2^		18 (40.9%)	39 (37.9%)	
Haematocrit, I/L			0.39 (0.27–0.50)	0.39 (0.24–0.52)	0.209
Haematocrit	Within normal range (F: 0.36–0.46 I/L, M: 0.4–0.5 I/L) ^2^		30 (68.2%)	69 (67.0%)	0.805
	Below normal range (F: <0.36 I/L, M: <0.4 I/L) ^2^		14 (31.8%)	33 (32.0%)	
	Above normal range (F: >0.46 I/L, M: >0.5 I/L) ^2^		0 (0.0%)	1 (1.0%)	
Mean corpuscular volume, fl			93.40 (85.10–109.00)	93.50 (63.40–115.70)	0.726
Mean corpuscular volume	Within normal range (F: 78.9–98.5 fl, M: 83.1–101.6 fl) ^2^		38 (86.4%)	80 (77.7%)	0.187
	Below normal range (F: <78.9 fl, M: <83.1 fl) ^2^		0 (0.0%)	7 (6.8%)	
	Above normal range (F: >98.5 fl, M: >101.6 fl) ^2^		6 (13.6%)	16 (15.5%)	
Mean corpuscular haemoglobin, pg			30.80 (27.20–33.70)	30.60 (16.10–40.20)	0.764
Mean corpuscular haemoglobin	Within normal range (F: 26.1–33.5 pg, M: 27.8–34.8 pg) ^2^		43 (97.7%)	86 (83.5%)	0.048 *
	Below normal range (F: <26.1 pg, M: <27.8 pg) ^2^		0 (0.0%)	9 (8.7%)	
	Above normal range (F: >33.5 pg, M: >34.8 pg) ^2^		1 (2.3%)	8 (7.8%)	
Mean corpuscular haemoglobin concentration, g/dL			32.40 (29.70–35.30)	32.40 (25.40–35.70)	0.944
Mean corpuscular haemoglobin concentration	Within normal range (F: 32.7–34.9 g/dL, M: 33–35 g/dL) ^2^		16 (36.4%)	47 (45.6%)	0.582
	Below normal range (F: <32.7 g/dL, M: <33 g/dL) ^2^		27 (61.4%)	54 (52.4%)	
	Above normal range (F: >34.9 g/dL, M: >35 g/dL) ^2^		1 (2.3%)	2 (1.9%)	
Red cell distribution width, %			13.55 (11.60–21.30)	14.00 (11.20–22.10)	0.143
Red cell distribution width	Within normal range (F: 12.4–17.3%, M: 12.1–16.3%) ^2^		35 (79.5%)	92 (89.3%)	0.254
	Below normal range (F: <12.4%, M: <12.1%) ^2^		5 (11.4%)	5 (4.9%)	
	Above normal range (F: >17.3%, M: >16.3%) ^2^		4 (9.1%)	6 (5.8%)	
Platelet count, ×10^9^/L			294.00 (163.00–490.00)	304.00 (101.00–808.00)	0.587
Platelet count	Within normal range (F: 186–454 × 10^9^/L, M: 171–388 × 10^9^/L) ^2^		41 (93.2%)	88 (85.4%)	0.398
	Below normal range (F: <186 × 10^9^/L, M: <171 × 10^9^/L) ^2^		1 (2.3%)	7 (6.8%)	
	Above normal range (F: >454 × 10^9^/L, M: >388 × 10^9^/L) ^2^		2 (4.5%)	8 (7.8%)	
Neutrophils, %			53.95 (18.10–81.40)	52.95 (19.90–79.10)	0.969
Neutrophil count, ×10^9^/L			2.55 (0.62–9.23)	2.54 (0.68–6.34)	0.952
Neutrophil count	Within normal range (F: 1.6–8.3 × 10^9^/L, M: 1.6–6.98 × 10^9^/L)^2^		32 (72.7%)	86 (84.3%)	0.05
	Below normal range (<1.6 × 10^9^/L)^2^		10 (22.7%)	16 (15.7%)	
	Above normal range (F: >8.3 × 10^9^/L, M: >6.98 × 10^9^/L)^2^		2 (4.5%)	0 (0.0%)	
Lymphocytes, %			33.30 (9.90–323.50)	34.90 (12.60–60.70)	0.765
Lymphocyte count, ×10^9^/L			1.63 (0.75–6.12)	1.65 (0.36–2.91)	0.389
Lymphocyte count	Within normal range (F: 1.4–4.5 × 10^9^/L, M: 1.4–4.2 × 10^9^/L)^2^		25 (56.8%)	72 (70.6%)	0.109
	Below normal range (<1.4 × 10^9^/L)^2^		18 (40.9%)	30 (29.4%)	
	Above normal range (F: >4.5 × 10^9^/L, M: >4.2 × 10^9^/L)^2^		1 (2.3%)	0 (0.0%)	
Monocytes, %			8.55 (3.90–15.70)	7.65 (4.10–25.50)	0.207
Monocyte count, ×10^9^/L			0.39 (0.20–1.28)	0.38 (0.15–0.93)	0.286
Monocyte count	Within normal range (F: 0.2–0.8 × 10^9^/L, M: 0.3–0.8 × 10^9^/L) ^2^		39 (88.6%)	97 (95.1%)	0.029 *
	Below normal range (F: <0.2 × 10^9^/L, M: <0.3 × 10^9^/L) ^2^		2 (4.5%)	5 (4.9%)	
	Above normal range (>0.8 ×10^9^/L) ^2^		3 (6.8%)	0 (0.0%)	
Eosinophils, %			2.00 (0.00–39.50)	2.45 (0.00–33.30)	0.316
Eosinophil count, ×10^9^/L			0.12 (0.00–1.39)	0.12 (0.00–1.52)	0.564
Eosinophil count	Within normal range (F: 0–0.4 × 10^9^/L, M: 0–0.95 × 10^9^/L) ^2^		41 (93.2%)	98 (96.1%)	0.431
	Above normal range (F: >0.4 × 10^9^/L, M: >0.95 × 10^9^/L) ^2^		3 (6.8%)	4 (3.9%)	
Basophils, %			0.50 (0.10–2.10)	0.60 (0.00–1.70)	0.471
Basophil count, ×10^9^/L			0.03 (0.01–0.30)	0.03 (0.00–0.07)	0.856
Basophil count	Within normal range (0–0.1 × 10^9^/L) ^2^		44 (100.0%)	102 (100.0%)	-
	Above normal range (>0.1 × 10^9^/L) ^2^		0 (0.0%)	0 (0.0%)	
Immature cells, %			0.30 (0.00–0.90)	0.30 (0.00–3.70)	0.884
Immature cell count, ×10^9^/L			0.02 (0.00–0.09)	0.02 (0.00–0.18)	0.623
CD45+ white cell count, ×10^9^/L			4.79 (2.35–11.31)	4.74 (2.67–7.93)	0.711
CD45+ white cell count	Within normal range (4–10 × 10^9^/L) ^2^		25 (59.5%)	75 (72.8%)	0.041 *
	Below normal range (<4 × 10^9^/L) ^2^		15 (35.7%)	28 (27.2%)	
	Above normal range (>10 × 10^9^/L) ^2^		2 (4.8%)	0 (0.0%)	
CD4 percentage of lymphocytes, %			13.28 (2.86–41.69)	14.57 (1.84–37.13)	0.965
CD4 percentage of lymphocytes	Within normal range (28–51%) ^2^		7 (16.7%)	4 (3.9%)	0.014 *
	Below normal range (<28%) ^2^		35 (83.3%)	99 (96.1%)	

^1^ Data retrieved from patient medical records reflecting last known result at time of recruitment. ^2^ Range as per NHLS definition. ^3^ Data collated from pharmacy records. Abbreviations: ART, antiretroviral therapy; HIV, human immunodeficiency virus; VL, viral load; FTC, Emtricitabine; EFV, Efavirenz; TDF, Tenofovir; 3TC, Lamivudine; DTG, Dolutegravir, AZT, Zidovudine; LPV/r, Lopinavir/Ritonavir; ABC, Abacavir; ATV/r, Atazanavir/Ritonavir; NVP, Nevirapine; TEE, TDF/FTC/EFV; TLD, TDF, 3TC, DTG; OD, optical density.

**Table 3:** Logistic regression for SARS-CoV-2 antibody response in HIV positive patients (*n* = 150). Model A includes biologically relevant demographic and clinical parameters as well as relevant parameters found to be associated with SARS-CoV-2 antibody response on a univariate level (see Table 2). Model B excludes parameters that were not significant in Model A.

Model A							
Characteristic	Unadjusted Odds Ratio	95% CI for Odds Ratio		Adjusted Odds Ratio	95% CI for Odds Ratio		*p*-Value
		Lower	Upper		Lower	Upper	
Age	0.998	0.962	1.036	1.008	0.965	1.052	0.721
Sex ^1^	1.235	0.582	2.617	0.785	0.323	1.908	0.593
ART ^2^	1.040	0.194	5.572	0.323	0.046	2.279	0.257
HIV VL control ^3^	2.667	1.019	6.977	2.915	1.035	8.210	0.043
CD45 positive white cell count ^4^	0.672	0.312	1.446	0.737	0.312	1.744	0.488
Mean corpuscular haemoglobin ^5^	0.000	0.000	0.000	0.000	0.000	0.000	0.999
Sodium ^6^	2.168	1.003	4.687	2.327	0.958	5.651	0.062
**Model B**							
**Characteristic**	**Unadjusted Odds Ratio**	**95% CI for Odds Ratio**		**Adjusted Odds Ratio**	**95% CI for Odds Ratio**		***p*-Value**
		**Lower**	**Upper**		**Lower**	**Upper**	
Age	0.998	0.962	1.036	1.001	0.962	1.041	0.975
Sex ^1^	1.235	0.582	2.617	0.831	0.364	1.893	0.659
ART ^2^	1.040	0.194	5.572	0.575	0.092	3.577	0.553
HIV VL control ^3^	2.667	1.019	6.977	2.961	1.078	8.133	0.035

^1^ Sex is for female compared to male. ^2^ ART is for “defaulted” compared to “receiving ART”. ^3^ HIV VL is for “not controlled” (VL > 1000 copies/mL) compared to VL < 1000 copies/mL. ^4^ CD45 is for “below normal range” compared to “within or above normal range”. ^5^ MCH is for “below normal range” compared to “within or above normal range”. ^6^ Sodium is for “below normal range” compared to “within normal range”.

## Data Availability

The data that support the findings of this article are openly available at PubMed (https://pubmed.ncbi.nlm.nih.gov/ accessed on 20 May 2022).

## Supplementary Materials

The following supporting information can be downloaded at: https://www.mdpi.com/article/10.3390/v14061222/s1, Figure S1: Detection of SARS-CoV-2 RBD- and S1-specific IgM and IgA antibodies in the study participants’ plasma; Table S1: Univariate analysis comparing the distribution of IgG, IgA and IgM responses to SARS-CoV-2 S1 and RBD antigens in the entire patient cohort (*n* = 150) based on the definition of IgG seroconversion, which was set at 2SD above the mean optical density of 30 pre-pandemic samples for each plate; Table S2: Univariate analysis comparing self-reported symptoms at presentation between patients who were negative (*n* = 44) and positive (*n* = 106) for SARS-CoV-2 antibodies. SARS-CoV-2 antibody positivity was detected by ELISA to IgG RBD and S1.
