# Supplementary material for: SARS-CoV-2 Infection Is Associated with Uncontrolled HIV Viral Load in Non-Hospitalized HIV-Infected Patients from Gugulethu, South Africa
Source: Viruses. 2022 Jun 3;14(6):1222. doi: 10.3390/v14061222 (PMC9229655; doi:10.3390/v14061222)
Supplement: Supplementary file 1 [file viruses-14-01222-s001.zip › viruses-1703287-supplementary.pdf]

viruses

MDPI

# Supplementary Materials

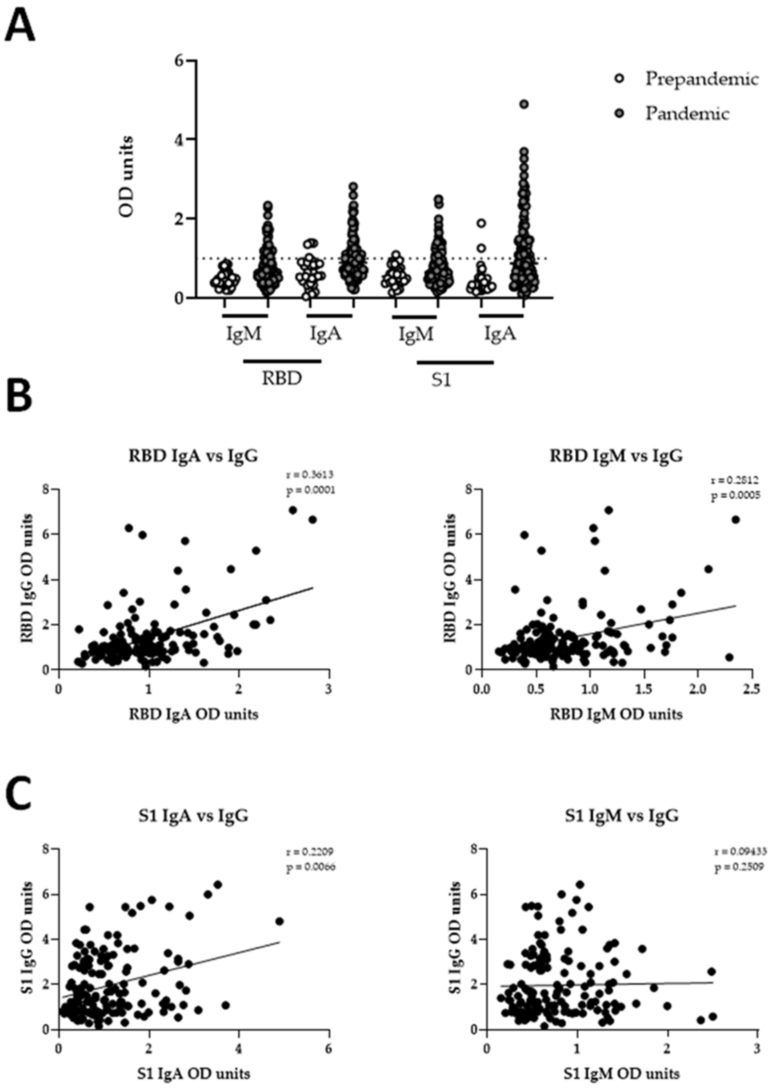


**Figure S1.** (**A**) Detection of SARS-CoV-2 RBD- and S1-specific IgM and IgA antibodies in the study participants' plasma. A total of thirty pre-pandemic patient samples [22] served as control. Results are represented by the OD units of each isotype, adjusted to the cut-off value of each individual plate and then normalized to the cut-off, which was set as one. The cut-off was determined by the mean OD + 2SD of the pre-pandemic samples. All samples were normalized to the cut-off value, which was set as one and is indicated by a dotted line. (**B**) The correlation between IgG and IgA (left panel) as well as IgG and IgM (right panel) responses to RBD SARS-CoV-2 antigens and (**C**) between IgG and IgA (left panel) as well as IgG and IgM (right panel) responses to S1 SARS-CoV-2 antigens. Statistical analyses were performed using a non-parametric Spearman Rank correlation.

**Table S1.** Univariate analysis comparing the distribution of IgG, IgA and IgM responses to SARS-CoV-2 S1 and RBD antigens in the entire patient cohort (*n* = 150) based on the definition of IgG seroconversion, which was set at 2SD above the mean optical density of 30 pre-pandemic samples [22] for each plate. All OD values to IgM and IgA RBD and S1 were normalized to plate cut-off and defined as positive if >2SD mean optical density of the pre-pandemic samples. Data are presented as number and percentage of total or median and range as appropriate. *P*-values are by Mann–Whitney test for continuous variables or Fisher's Exact test for categorical variables as appropriate. $p < 0.05$ is considered significant and indicated with *.

| | | SARS-CoV-2 Antibody Negative (*n* = 44) N (%) or Median (Range) | SARS-CoV-2 Antibody Positive (*n* = 106) N (%) or Median (range) | *p*-Value |
|---|---|---|---|---|
| RBD IgG, OD | | 0.71 (0.16–0.96) | 1.25 (0.47–7.09) | <0.001 * |
| RBD IgG | negative | 44 (100.0%) | 30 (28.3%) | <0.001 * |
| | positive | 0 (0.0%) | 76 (71.7%) | |
| RBD IgM, OD | | 0.57 (0.15–2.29) | 0.67 (0.18–2.34) | 0.032 * |
| RBD IgM | negative | 38 (86.4%) | 75 (70.8%) | 0.043 * |
| | positive | 6 (13.6%) | 31 (29.2%) | |
| RBD IgA, OD | | 0.74 (0.21–1.98) | 0.93 (0.22–2.82) | 0.002 * |
| RBD IgA | negative | 34 (77.3%) | 59 (55.7%) | 0.013 * |
| | positive | 10 (22.7%) | 47 (44.3%) | |
| S1 IgG, OD | | 0.72 (0.15–0.98) | 1.83 (0.29–6.44) | <0.001 * |
| S1 IgG | negative | 44 (100.0%) | 8 (7.5%) | <0.001 * |
| | positive | 0 (0.0%) | 98 (92.5%) | |
| S1 IgM, OD | | 0.56 (0.20–2.50) | 0.67 (0.15–2.49) | 0.028 * |
| S1 IgM | negative | 36 (81.8%) | 75 (70.8%) | 0.16 |
| | positive | 8 (18.2%) | 31 (29.2%) | |
| S1 IgA, OD | | 0.64 (0.08–3.09) | 1.08 (0.11–4.90) | <0.001 * |
| S1 IgA | negative | 36 (81.8%) | 49 (46.2%) | <0.001 * |
| | positive | 8 (18.2%) | 57 (53.8%) | |

**Table S2.** Univariate analysis comparing self-reported symptoms at presentation between patients who were negative (*n* = 44) and positive (*n* = 106) for SARS-CoV-2 antibodies. SARS-CoV-2 antibody positivity was detected by ELISA to IgG RBD and S1 ("Negative" indicates that a patient sample had an OD value below the assay cut-off for both IgG RBD and S1 ELISAs). Data are presented as number and percentage of total. Participants with missing data are excluded per characteristic. *p*-values are by Fisher's Exact test. $p < 0.05$ is considered significant.

| | | SARS-CoV-2 Antibody Negative (*n* = 44) N (%) or Median (Range) | SARS-CoV-2 Antibody Positive (*n* = 106) N (%) or Median (Range) | *p*-Value |
|---|---|---|---|---|
| Other co-infection | No | 42 (95.5%) | 99 (97.1%) | 0.638 |
| | Yes | 2 (4.5%) | 3 (2.9%) | |
| Fever | No | 44 (100.0%) | 103 (98.1%) | 1.00 |
| | Yes | 0 (0.0%) | 2 (1.9%) | |
| Fatigue | No | 43 (97.7%) | 101 (96.2%) | 1.00 |
| | Yes | 1 (2.3%) | 4 (3.8%) | |
| Edema | No | 42 (95.5%) | 100 (96.2%) | 1.00 |
| | Yes | 2 (4.5%) | 4 (3.8%) | |
| Cachexia | No | 42 (97.7%) | 99 (96.1%) | 1.00 |
| | Yes | 1 (2.3%) | 4 (3.9%) | |
| Respiratory symptoms | No | 44 (100.0%) | 95 (94.1%) | 0.178 |
| | Yes | 0 (0.0%) | 6 (5.9%) | |

| | | | | |
|---|---|---|---|---|
| Gastrointestinal disturbance | No | 42 (97.7%) | 101 (98.1%) | 1.00 |
| | Yes | 1 (2.3%) | 2 (1.9%) | |
| Arthralgia | No | 40 (97.6%) | 87 (94.6%) | 0.666 |
| | Yes | 1 (2.4%) | 5 (5.4%) | |
| Altered mental state | No | 43 (97.7%) | 102 (97.1%) | 1.00 |
| | Yes | 1 (2.3%) | 3 (2.9%) | |
| Neuoropathy | No | 42 (100.0%) | 102 (97.1%) | 0.558 |
| | Yes | 0 (0.0%) | 3 (2.9%) | |
| Radiographic abnormalities | No | 44 (100.0%) | 104 (100.0%) | 1.00 |
| | Yes | 0 (0.0%) | 0 (0.0%) | |
| Coughing | No | 42 (100.0%) | 98 (93.3%) | 0.192 |
| | Yes | 0 (0.0%) | 7 (6.7%) | |
| Loss of weight | No | 40 (95.2%) | 95 (90.5%) | 0.510 |
| | Yes | 2 (4.8%) | 10 (9.5%) | |
| Night sweats | No | 41 (95.3%) | 98 (93.3%) | 1.00 |
| | Yes | 2 (4.7%) | 7 (6.7%) | |
| Overall symptoms at presentation | No | 39 (88.6%) | 80 (75.5%) | 0.079 |
| | Yes | 5 (11.4%) | 26 (24.5%) | |
